# Aerodynamic dataset for selected doubly curved membrane canopy structures

**DOI:** 10.1038/s41597-025-06046-w

**Published:** 2025-10-08

**Authors:** Anoop Kodakkal, Kimberly Adamek, Ann-Kathrin Goldbach, Tibebu Birhane, Rodrigo Castedo-Hernandez, Guillermo Martínez-López, Máté Péntek, Kai-Uwe Bletzinger, Roland Wüchner, Girma Bitsuamlak

**Affiliations:** 1 Chair of Structural Analysis, Technical University of Munich, Munich, Germany; 2 WindEEE Research Institute, Western University, London, Ontario Canada; 3 Universitat Politècnica de València, Valencia, Spain

**Keywords:** Civil engineering, Databases

## Abstract

A dataset of aerodynamic measurements is collected for doubly curved membrane structures as part of a comprehensive experimental testing campaign on wind effects on structural membranes conducted at the WindEEE Dome at Western University, Canada. Common doubly curved membrane geometries – the hypar, ridge valley, arch supported, cone, and umbrella – were tested in isolated instances. The cone geometry was also tested in both a 1 × 3 row and a 3 × 3 group arrangement. All models were tested at a 1:25 scale under atmospheric boundary layer (ABL) flow at angles of attack ranging from 0° to 180° in 10° increments (and 45°, and 135° -depending on the line of symmetry). In addition to ABL, the hypar geometry was subjected to two other distinct flow scenarios: tornado, and downburst. Pressure time series at various tap locations are included in the data. In total, approximately 425 tests were conducted, providing a comprehensive dataset on the aerodynamic behavior of doubly curved structures under wind loads. This experimental data set offers valuable insights for the design and analysis of such structures in architectural and engineering applications and future design guideline developments. Data is available on an open-source dataset Zenodo as part of the ERIES-WENSS project.

## Background & Summary

Prestressed membrane structures are widely favored in public spaces across various scales due to their architectural appeal and force-efficient geometry. These structures can cover large areas with minimal material use, making them a resource-efficient choice. Structural engineers and architects are responsible for determining the mechanically optimal shapes, while fabric manufacturers supply ultra-light materials capable of withstanding high levels of tensile forces. Tensile structures such as mechanically prestressed membranes require a double-curved shape in order to achieve sufficient strength and stability to withstand external loading. The low structural mass combined with large surface coverage often leads to scenarios characterized by added-mass effects and potential fluid-structure interactions. However, wind loading patterns on these structures are often only approximations, with only a few specialized projects receiving dedicated wind tunnel or numerical analysis.

Typical shapes are found in cable net structures and membrane canopies, where the geometry is determined in a formfinding analysis for a given prestress and boundary conditions. This study is inspired by several iconic examples of freestanding canopies, such as the Tanzbrunnen in Köln (1957, membrane), the Olympic Stadium Roof in Munich (1972, cable net), and the inverted umbrellas in the Haram Plaza in Medina (2010, deployable membranes). These landmark projects have driven significant engineering advancements. Despite such constructions being widespread, the critical load case of wind on relatively large surfaces (to the structure) has not been sufficiently studied. More precisely, there is a major lack of codification and standardization on the wind effect of these structures, mainly due to their unique shapes that require special studies.

**Fig. 1:**
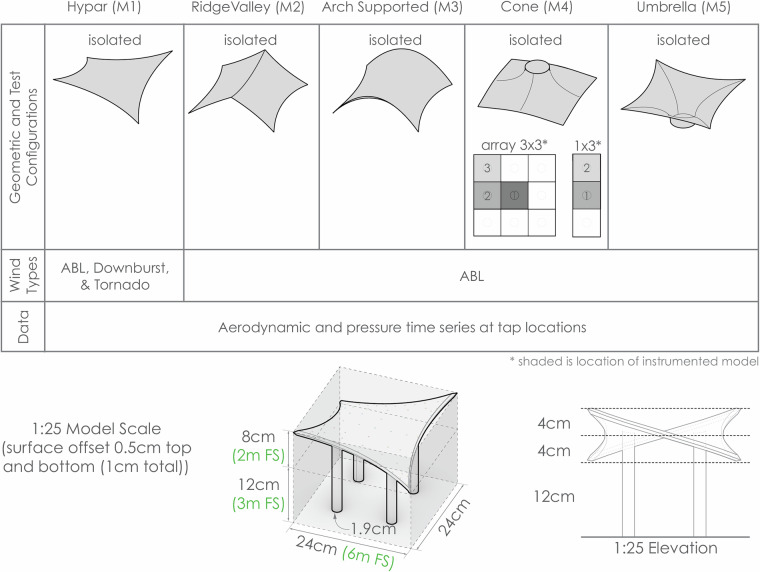
Schematic overview of the aerodynamic wind data set for non-standard shapes and structures. FS refers to full scale.

The present study examines five generic shapes–hypar, ridge valley, arch supported, cone and umbrella, as illustrated in Fig. 1^1^–which have been identified as representative in literature^2^ and the JRC Science and Policy Report on Tensile Membrane Structures^3,4^. These shapes were specifically highlighted in the 2015 Round Robin 3 (RR3) call (TensiNet, 2015) and its subsequent findings^5^. The focus of the study is to present high-resolution pressure map time series data, which can be utilized by practicing engineers and scientists in similar design scenarios. Together with isolated instances, various group configurations are tested in the wind tunnel under atmospheric boundary layer conditions. Additionally, the hypar geometry is investigated not only under atmospheric boundary layer flow but also under non-synoptic wind loads such as tornado and downburst, in order to investigate the phenomenological distinctions of the different wind events. A brief description of the data set, including the various geometries tested, configurations, and loading scenarios, is shown in Fig. 1. This extensive dataset can serve various purposes, including validation studies as in^6,7^, conducting numerical simulations, and more. The dataset supports engineers and practitioners in designing membrane structures with typical doubly curved shapes. It provides a foundation for professionals working not only with atmospheric boundary layer flows but also with other extreme types of wind flows. The dataset for downbursts and tornadoes can also be beneficial for computational fluid dynamics and natural catastrophe modeling communities.

The reported geometry scale is 1:25. In this dataset, around 425 measurements are recorded under multiple wind conditions for the shapes mentioned and for the group of configurations as in Fig. 1. The pressure time series at the tap locations are presented for all the configurations at Bletzinger et al 2024^8^.

## Methods

### Wind Engineering, Environment, and Energy (WindEEE) research facility

**Fig. 2:**
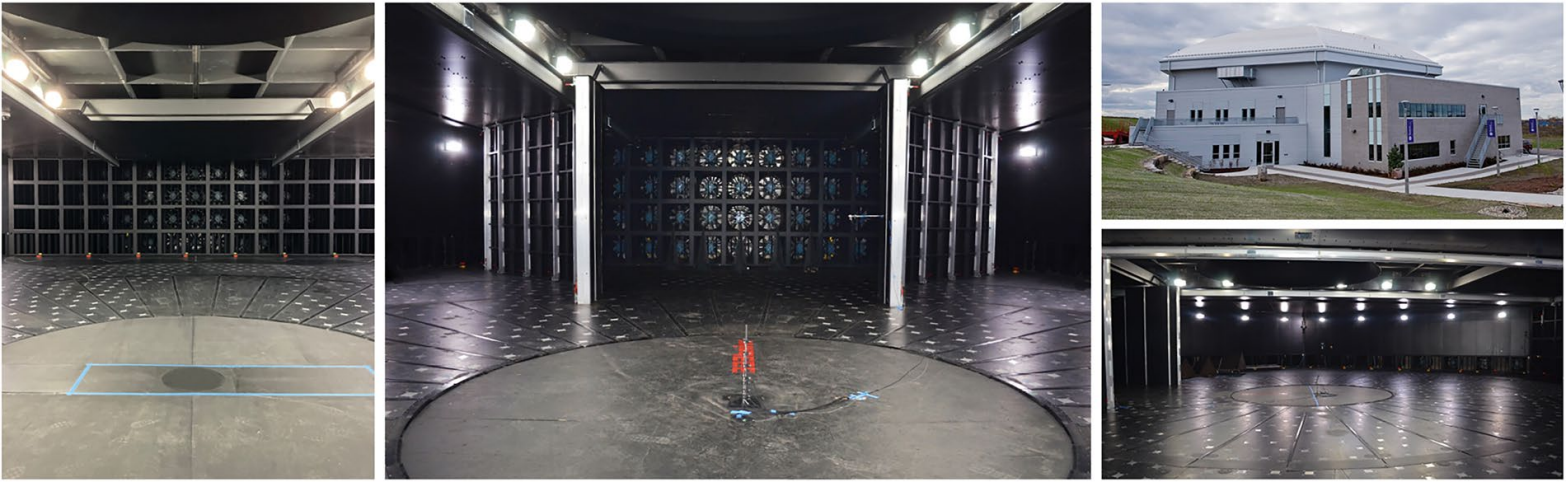
Details of the WindEEE Dome. Fan wall inside WindEEE Dome (left); Contraction wall (middle); The WindEEE Dome building in London, Ontario (right top); and Sideview inside WindEEE Dome (right bottom).

**Fig. 3:**
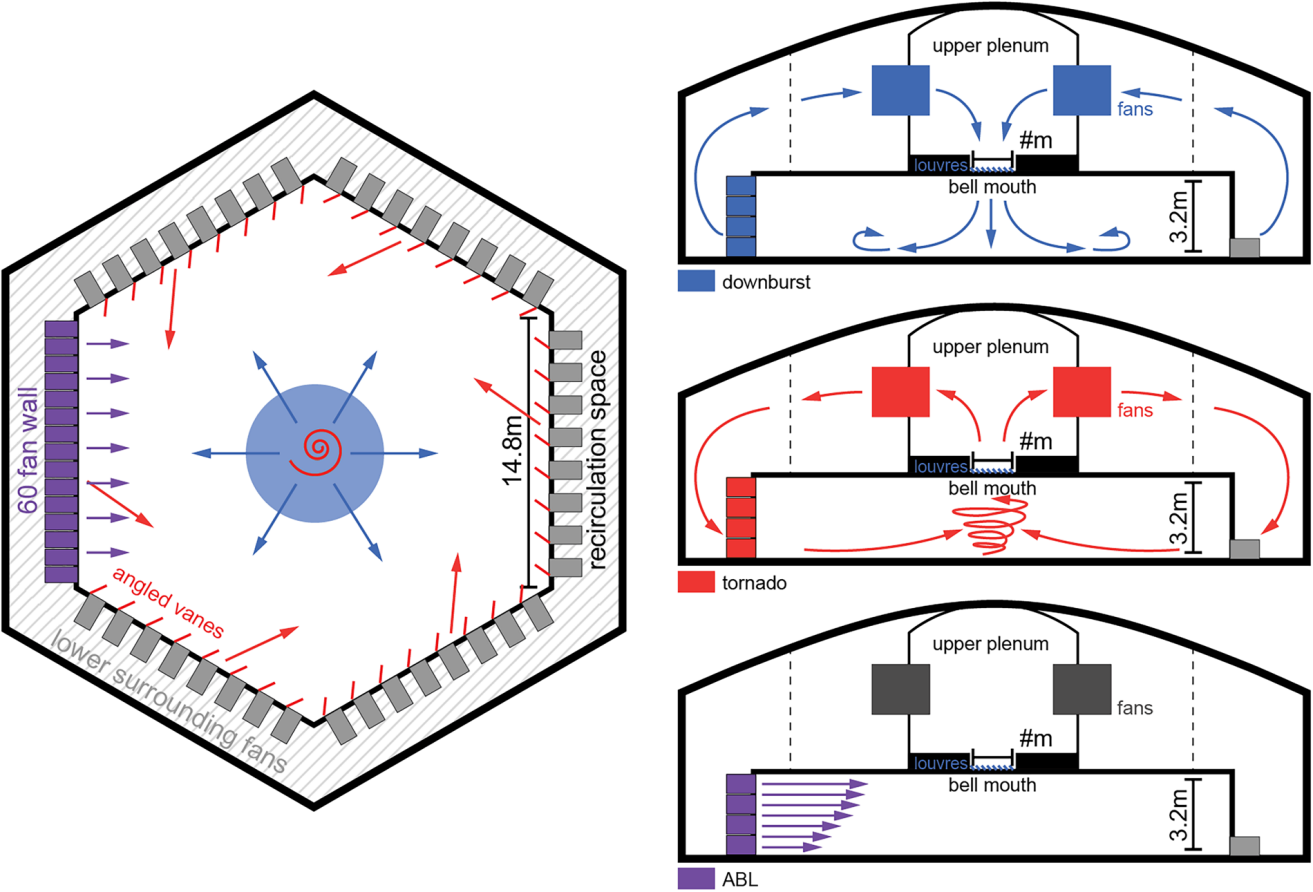
WindEEE chamber flow generation diagrams; plan view (left); sectional views (right).

All experimental tests, including the ABL, downburst, and tornado characterization and membrane interaction experiments outlined in this research were performed at the WindEEE Dome. The WindEEE Dome, at Western University in London, Ontario has the unique capability to replicate boundary layer, shear, downburst, and tornadic-like wind flows. Details of the test facility are shown in Figs. 2 and 3.

### Wind field instrumentation and characterization

**Fig. 4:**
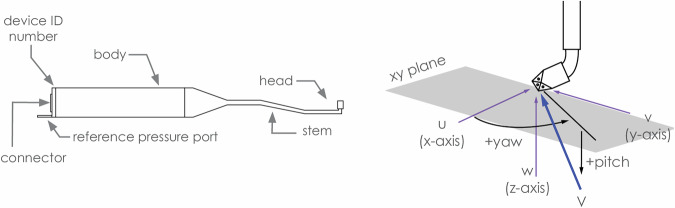
Diagram of ‘j-shaped’ 100 series cobra probe device (left); flow axis system with respect to the probe head and positive flow pitch and yaw angles (Getting Started - Cobra Probe) (right).

A turbulent boundary layer flow was identified and developed whose mean wind speed and longitudinal turbulence profile fit target atmospheric boundary layer (ABL) profiles at the length scale of 1:25. All five pressure models were tested under the simulated ABL flow. Moreover, the hypar model was tested under downburst and tornado winds. The three components of the flow velocity were measured using Turbulent Flow Instrumentation (TFI)’s Cobra probes. The direction of the u-, v- and w- components relative to the probe heads are shown in Fig. 4. The cobra probes have a cone of capture which allows them to sample wind velocity within +/−45 degrees from the point of its nozzle. Probe information, including the specifications of TFI products and calculations the probes perform, can be found online at Turbulent Flow Instrumentation.

#### ABL (Atmospheric Boundary Layer) flow

**Fig. 5:**
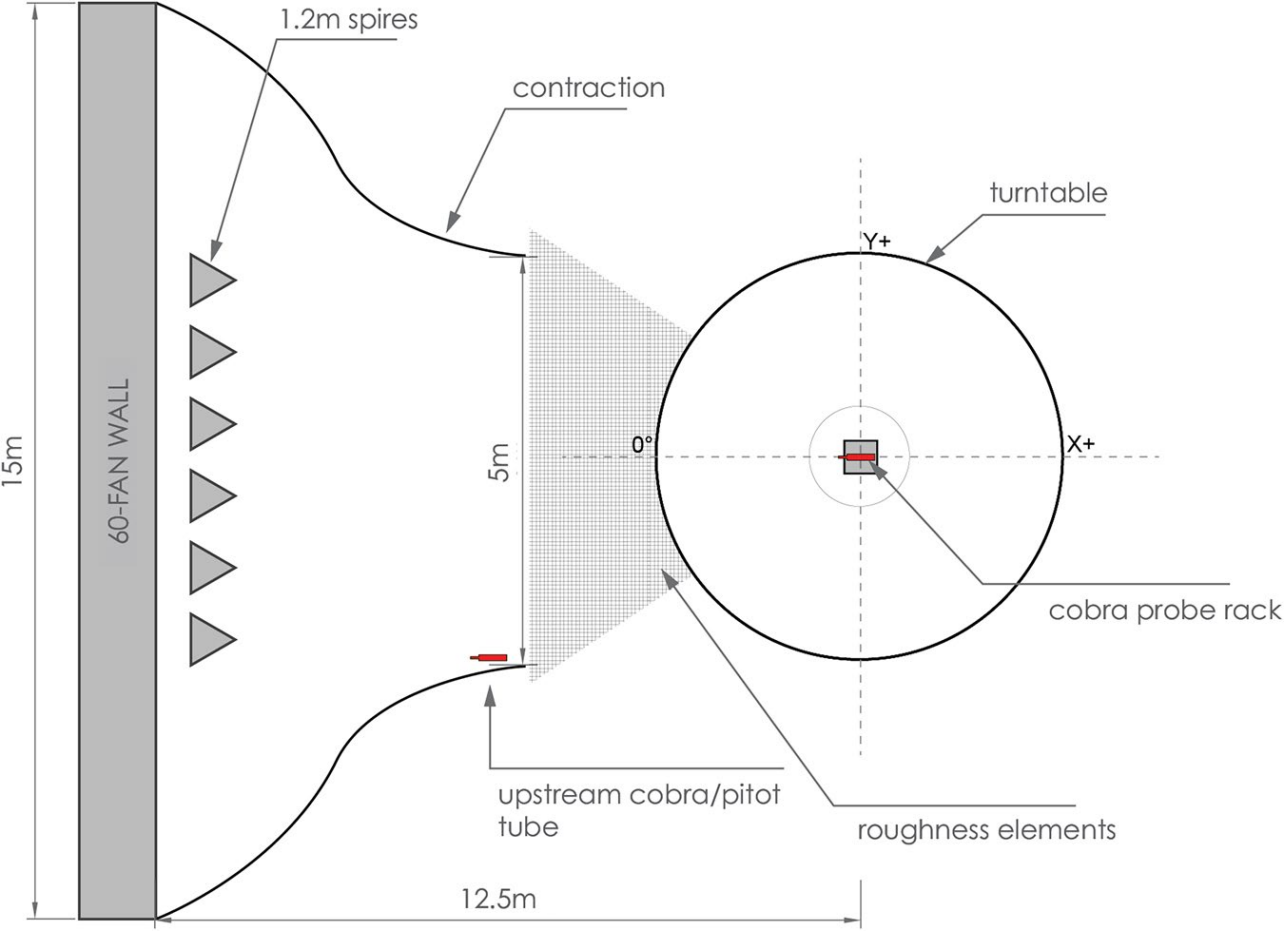
Details of the ABL wind generation and model testing at WindEEE.

**Fig. 6:**
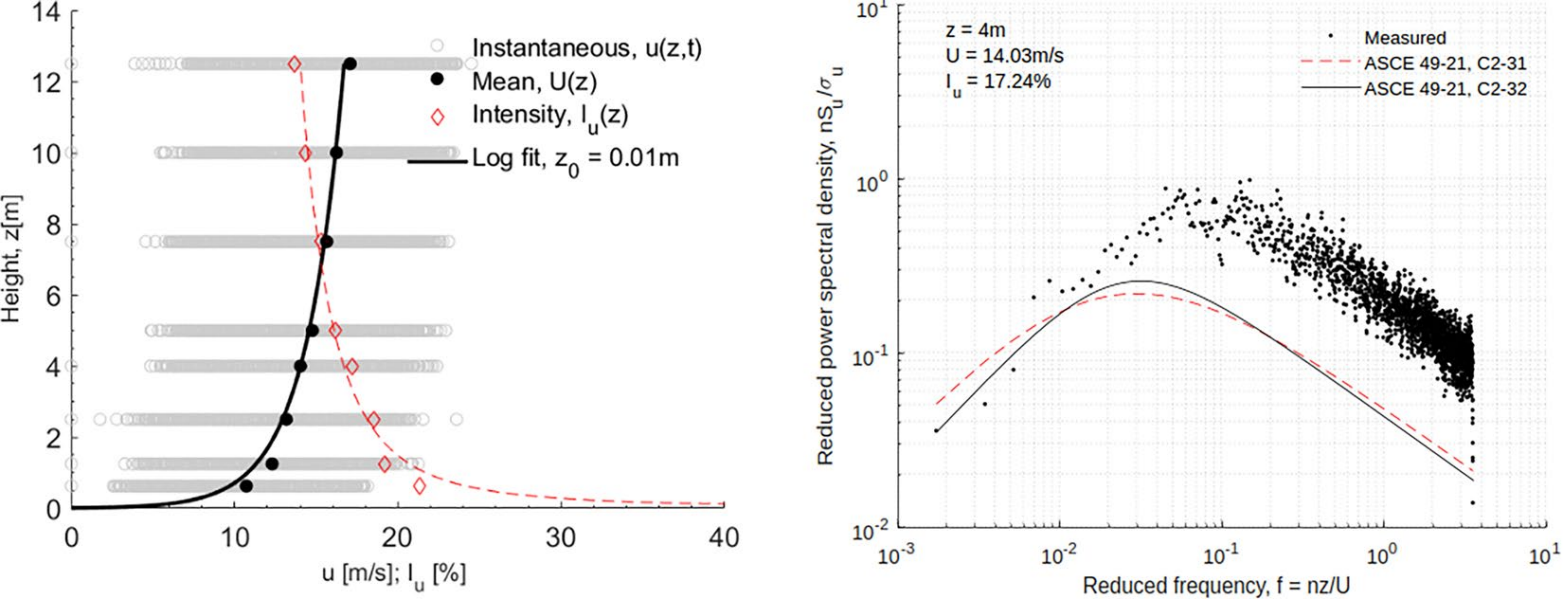
Mean velocity and TI profile measured with fitted profile (left). Spectra of the longitudinal wind turbulence at 16 cm height in model scale compared with ASCE 49-21 (right).

All the pressure models were tested under sheared turbulent boundary layer flow. The flow is driven by a matrix of 60 fans mounted on wall 1 of the test chamber, as shown in Fig. 5. The contraction wall, having a height equal to the ceiling height of the test chamber and a width reducing from 15 m to 5 m, is used to increase the wind speed at the center of the turntable. The mean flow shear and the turbulence intensity of the simulated ABL flow are controlled by the triangular spires, the inlet to the test chamber, and the floor roughness similar to^9^. The simulated ABL flow for testing pressure models was generated by using triangular spires of 123 cm tall, 40 cm wide, and with center-to-center spacing of 80 cm. Figure 6 shows the vertical profile of the mean flow wind speed and the longitudinal turbulence. The spectra of the longitudinal wind turbulence at the mean roof height of the tested pressure models are also shown on the left.

#### Downburst outflow wind

**Fig. 7:**
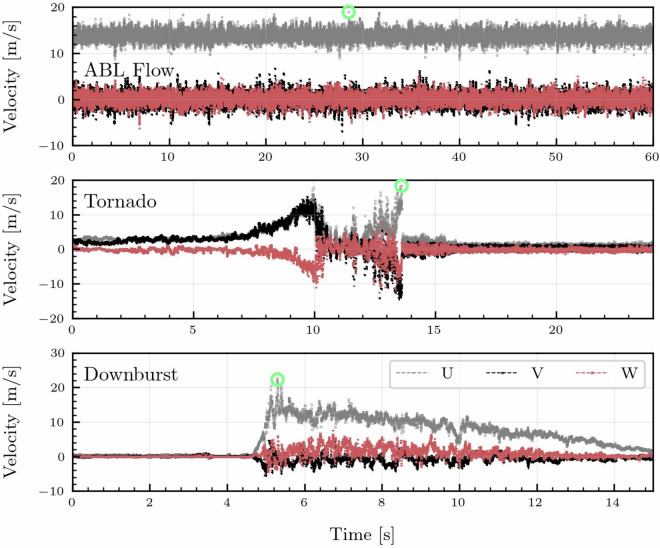
Time history of velocity in the three directions for ABL, tornado, and downburst measured by the reference velocity probe during pressure testing of the hypar model.

Downburst flow is generated at the WindEEE dome through the use of six large fans above the testing chamber, pressurizing the upper plenum and then releasing this pressure through the bell mouth into the testing chamber below. By utilizing different pressures, bellmouth opening sizes, and opening speeds, a downdraft velocity produces a downburst in the test chamber. The WindEEE Dome test chamber can generate many sizes of downbursts through the manipulation of input parameters such as the downdraft diameter, Dbm ; upper fan speed, Puf (alters the differential pressure between upper and main chambers); and manipulating the time sequence of fan rpms and opening of the bellmouth louvers. During the research outlined in this study, the WindEEE dome bell mouth was set to a diameter of 3.2 m, upper fan speed set to 40%. Figure 7 (bottom) shows a typical time history of the downburst outflow velocity at the reference probe. Details of the wind speed profiles as a function of height above the floor and radial distance from the center of the downdraft jet can be found in the work of Chowdhury 2018^10^.

#### Tornado wind

The wide dome is capable of generating tornado vortices with core diameters ranging from 50 cm to 240 cm. The flow speed and structure of the vortices are controlled by the rpm of the six large fans in the upper plenum, the diameter of the updraft hole between the test chamber and the upper plenum, the louver angles at the inlet of the test chambers, and the test chamber floor roughness. The hypar model was tested under the action of two tornado vortices having the radius of maximum winds equal to 60 cm and 45 cm at height of 20 cm above the floor (see ^11^). These vortices were generated by running the upper fans at  −50% of their rated rpm, setting the inlet louver angles to 15 and 25 degrees, and the updraft hole size of 320 cm. In both simulation cases, the depth of the inflow to the 2500 cm diameter test chamber equals to 80 cm. Figure 7 (middle) shows a typical time history of the flow velocity at the reference probe during the pressure testing of the hypar model.

### Aerodynamic pressure testing

#### Instrumentation

**Fig. 8:**
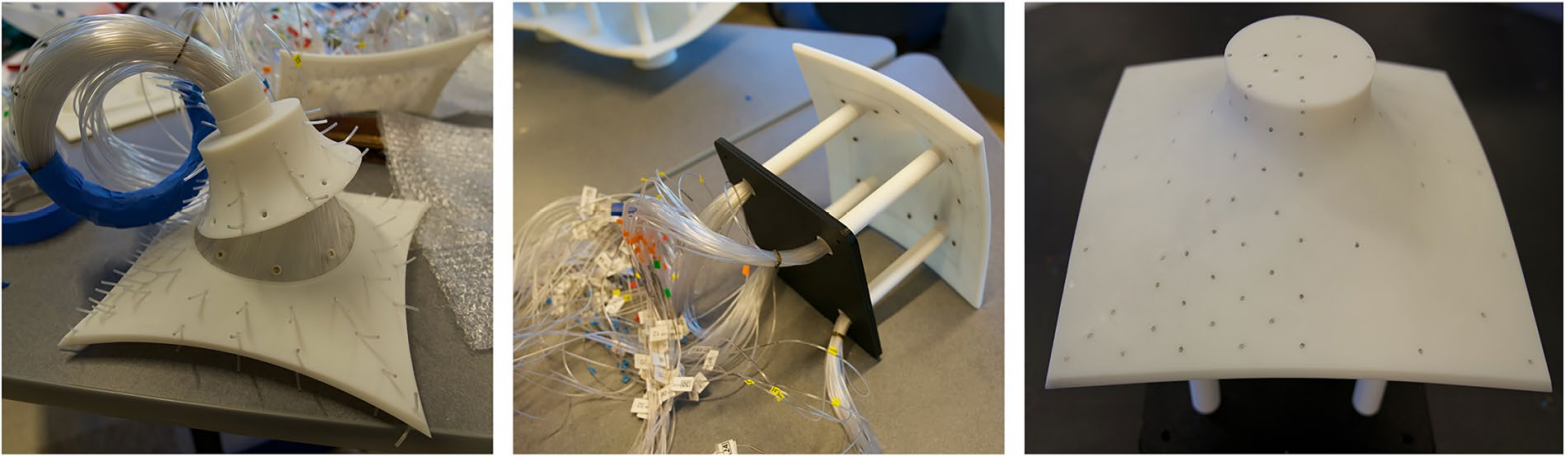
Pressure system instrumentation details on models.

The models are instrumented with tubes or ‘taps’ that are attached to pressure scanners which are connected to a data acquisition system. A bag test and a puff test are performed on each model prior to testing to ensure all pressure taps are functioning and to document which are not. The pressure models are connected to the ESP pressure scanners using flexible PVC tubing 40 inches (approximately 1.02 meters) in length. The dynamic effect of the tubing on the measured pressure is compensated by applying the aerodynamic pneumatic admittance function of the tubing system. Each scanner supports 32 pressure ports hooked up to the models. Figure 8 shows the tubes connected to various models during the model preparation phase.

#### Hypar (M1; hyperbolic paraboloid) pressure model

The Hypar (M1) doubly curved geometry is tested at a scale of 1:25 under ABL, Downburst, and Tornado winds. The equilibrium shape of the M1 hypar can be determined via formfinding for a prestress ratio of 4 (e.g. kN/m) as an isotropic membrane prestress to 30 (e.g. kN) in the edge cables, with fixed support points. The full-scale size of the geometry is 6 × 6 × 5 m, and at a scale of 1:25, the tested model is 24 × 24 × 20 cm, where the canopy itself is 8 cm tall, beginning 12 cm from the ground. The model is 1 cm thick overall (offset 0.5 cm above and below the original surface geometry) with a 0.6 cm cavity in between for pressure tubing.

**Fig. 9:**
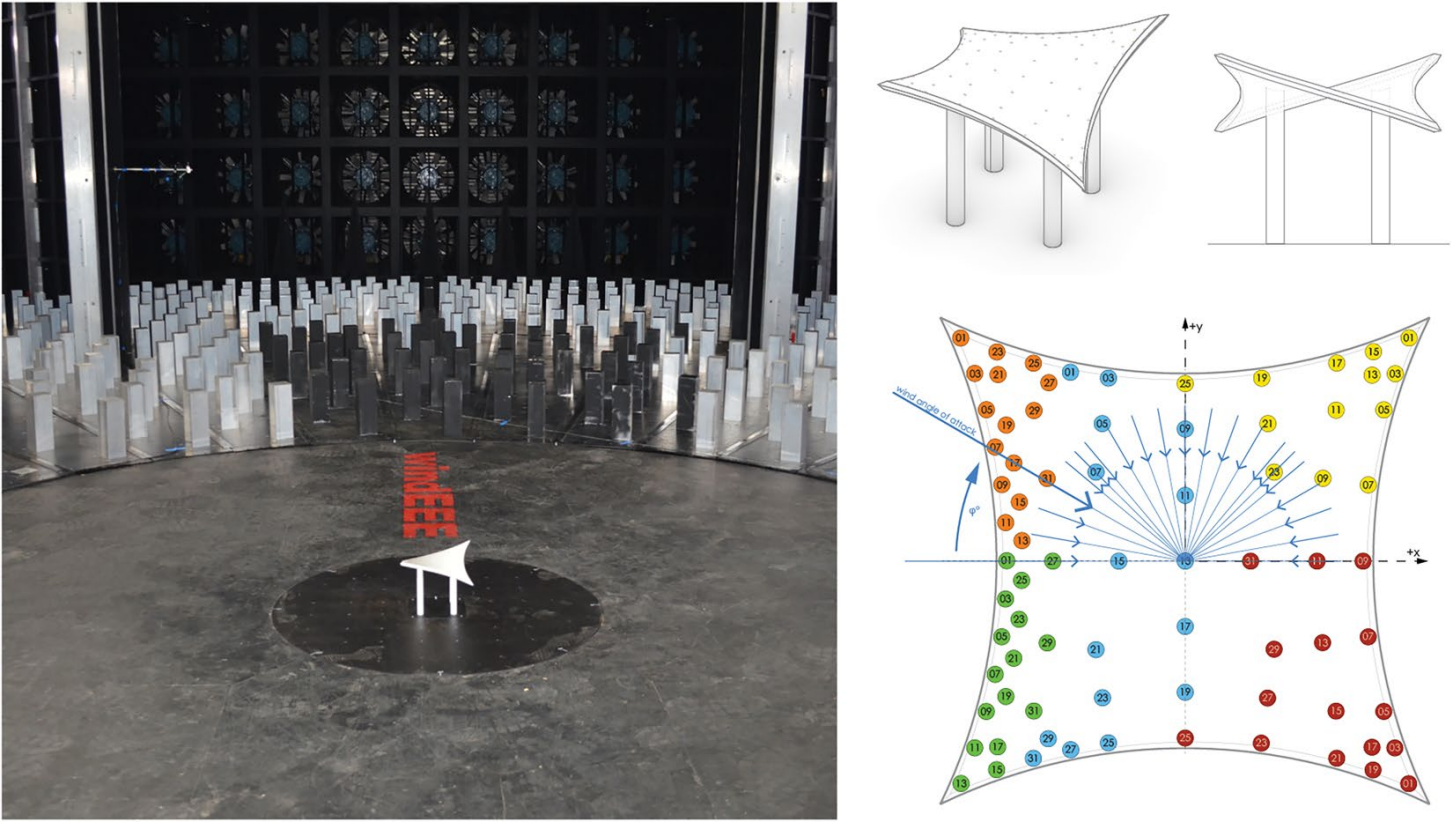
Hypar (M1) sample tap layout, 3D image and elevation (colors represent scanner numbers - further details available at^8^).

The model was instrumented with approximately 154 pressure taps as shown in Fig. 9. Pressure scanners were connected to the instrumented tap locations on the membrane structure. Pressure tap coordinates are available in the data set to correlate instantaneous measurements with locations covering the model. The taps are evenly distributed across the top and bottom of the entire model. Additional taps are located on the leading-edge of the model (−90° to 90°) to gather additional data along the symmetry plane. Upstream velocity measurements at the reference location were captured using one ‘Straight’ TFI Cobra Probe.

##### ABL loading

This test involved the model described above subjected to low-speed ABL, and high-speed ABL. The model was tested every 10° between 0 and 180° plus a 45° and 135° angle of attack.

##### Downburst loading

**Fig. 10:**
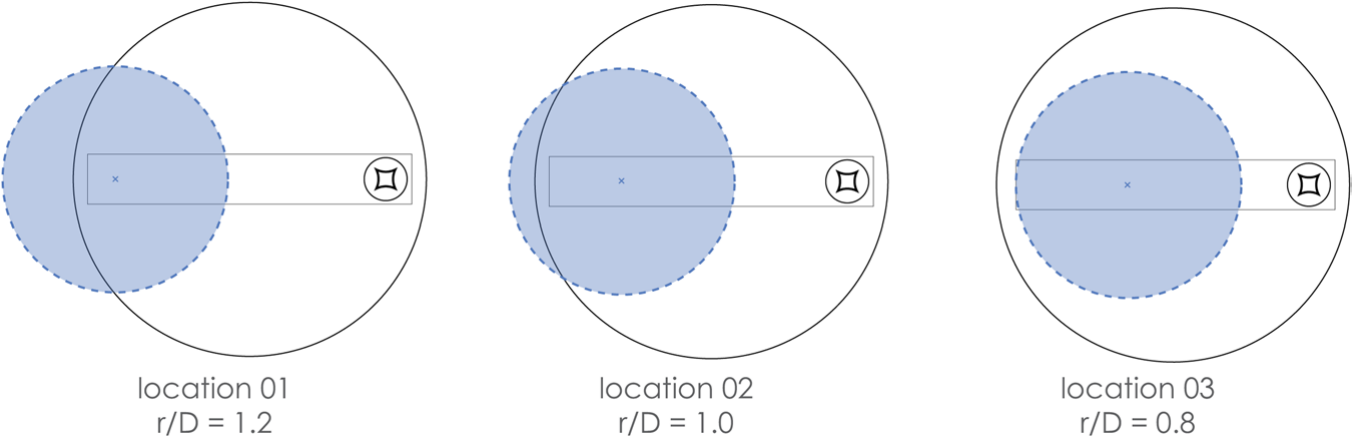
Downburst configurations tested.

This test involved the M1 Hypar model tested under downburst-like flow loading conditions in three configurations where the model is offset from the center of the turntable to achieve 3 r/D values: 0.8, 1.0, and 1.2 (in the -x direction) (offset 0.65 m, 1.29 m and 1.95 m respectively) as shown in Fig. 10.

**Fig. 11:**
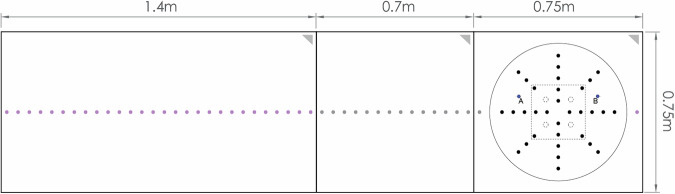
Ground floor pressure tap layouts.

In addition to the instrumented M1 model, 32 ground taps were located adjacent to the study model arranged in lines at 0°, −45°, 45°, and 90° axes. An additional 44 taps were arranged in a line along the ground at 0° on either side of the model to capture the downburst-like flow fields overall. Two J-probes were also located on the ground near the front edge and back edge of the model at a height of 16 cm (the average roof height of the structure) as shown in Fig. 11.

##### Tornado loading

**Fig. 12:**
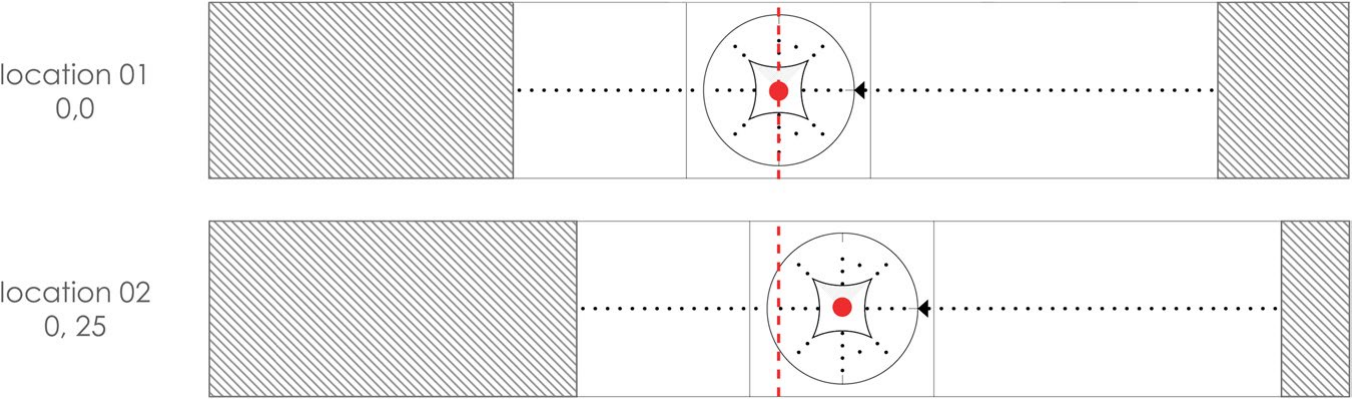
Tornado configurations tested.

This test involved the Hypar (M1) model tested under tornado-like flow loading conditions in two configurations where the model is offset from the center of the turntable by 0 cm and 25 cm (in the -x direction) as shown in Fig. 12.

The same instrumented Hypar model and ground taps, as described in the downburst test, were employed for the tornado flow.

#### Ridge valley (M2) pressure model

The purpose of this model was to test the ridge valley (M2) doubly curved geometry at a scale of 1:25 under ABL winds. The equilibrium shape of the ridgevalley (M2) can be determined via formfinding for a prestress ratio of 4 (e.g. kN/m) as an isotropic membrane prestress to 30 (e.g. kN) in the edge and ridge cables, with fixed support points. The full-scale size of the geometry is 6 × 6 × 5 m, and at a scale of 1:25, the tested model is 24 × 24 × 20 cm, where the canopy itself is 8 cm tall beginning 12 cm from the ground. The canopy is 1 cm thick overall (offset 0.5 cm above and below the original surface geometry) with a 0.6 cm cavity in between for pressure tubing.

**Fig. 13:**
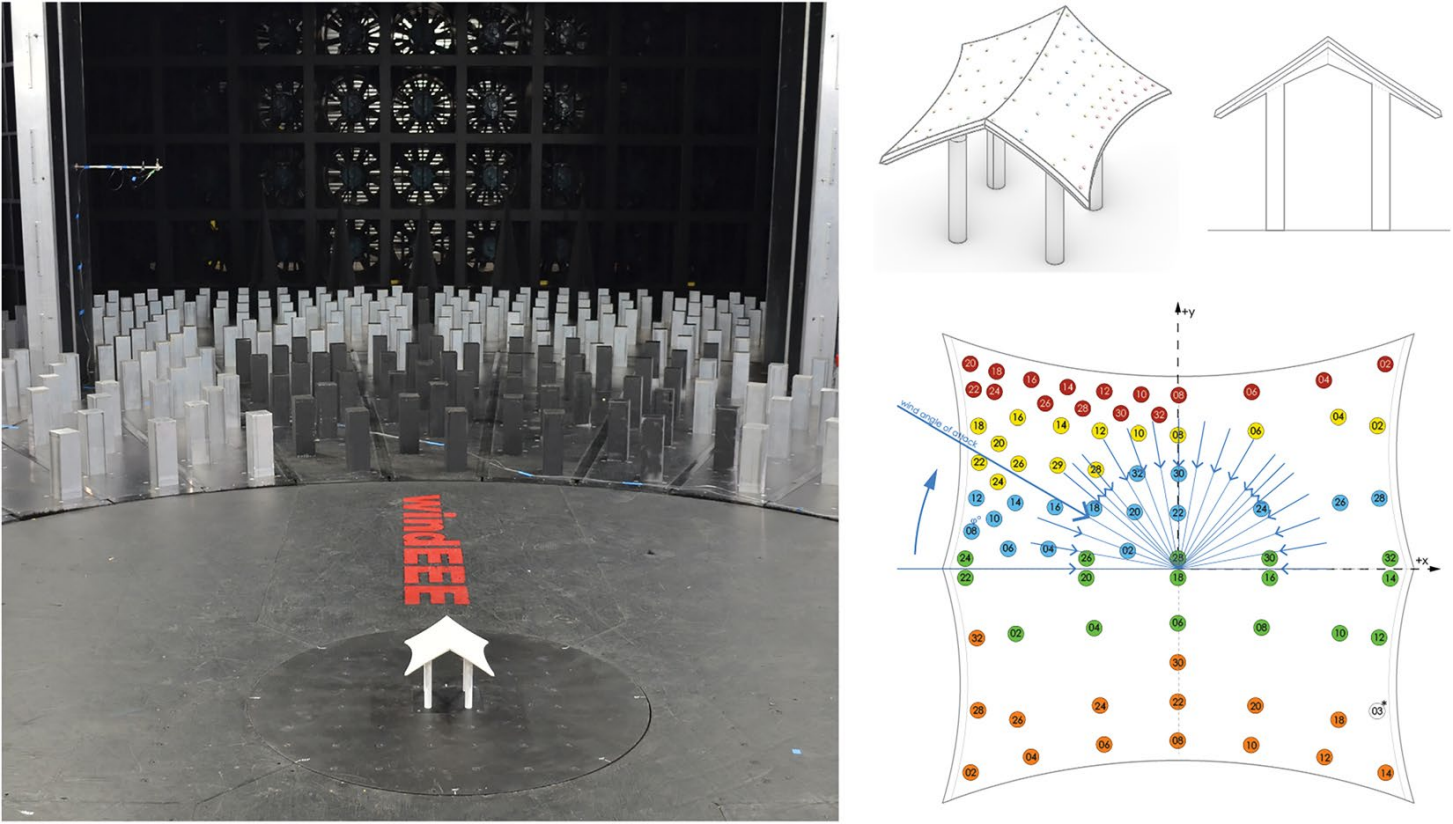
Ridge valley (M2) sample tap layout, 3D image and elevation (colors represent scanner numbers - further details available at^8^).

The model was instrumented with approximately 157 pressure taps as shown in Fig. 13. Pressure scanners were connected to the instrumented tap locations on the membrane structure. Pressure tap coordinates are available to correlate instantaneous measurements with locations covering the model in the data set. The taps are evenly distributed across the top and bottom of the entire model with extra taps located on the leading-edge quadrant of the model (0° to 90°) to gather additional data along the symmetry plane. The top and bottom taps are directly above/below one another in the z direction, with even tap numbers on top and odd numbers on the bottom following the same order. Upstream velocity measurements were captured using one ‘Straight’ TFI Cobra Probe at the reference point.

##### ABL loading

This test involved the model described above subjected to low-speed ABL, and high-speed ABL. The model was tested every 10° between 0 and 180°, together with 45° and 135° angles of attack.

#### Arch supported (M3) pressure model

The purpose of this specimen was to test the arch supported (M3) doubly curved geometry at a scale of 1:25 under ABL winds. The equilibrium shape of the M3 can be determined via formfinding for a prestress ratio of 4 (e.g. kN/m) as an isotropic membrane prestress to 30 (e.g. kN) in the edge cables, with fixed line supports at the arches. The arch geometry is defined as a NURBS curve (p = 3, CPs at (0,0,0), (0,2,2.65), (0,4,2.65), (0,6,0), knot vector (0,0,0,1,1,1)). The full-scale size of the geometry is 6 × 6 × 5 m and at a scale of 1:25 the tested model is 24 × 24 × 20 cm where the canopy itself is 8 cm tall beginning 12 cm from the ground. The canopy is 1 cm thick overall (offset 0.5 cm above and below the original surface geometry) with a 0.6 cm cavity in between for pressure tubing.

**Fig. 14:**
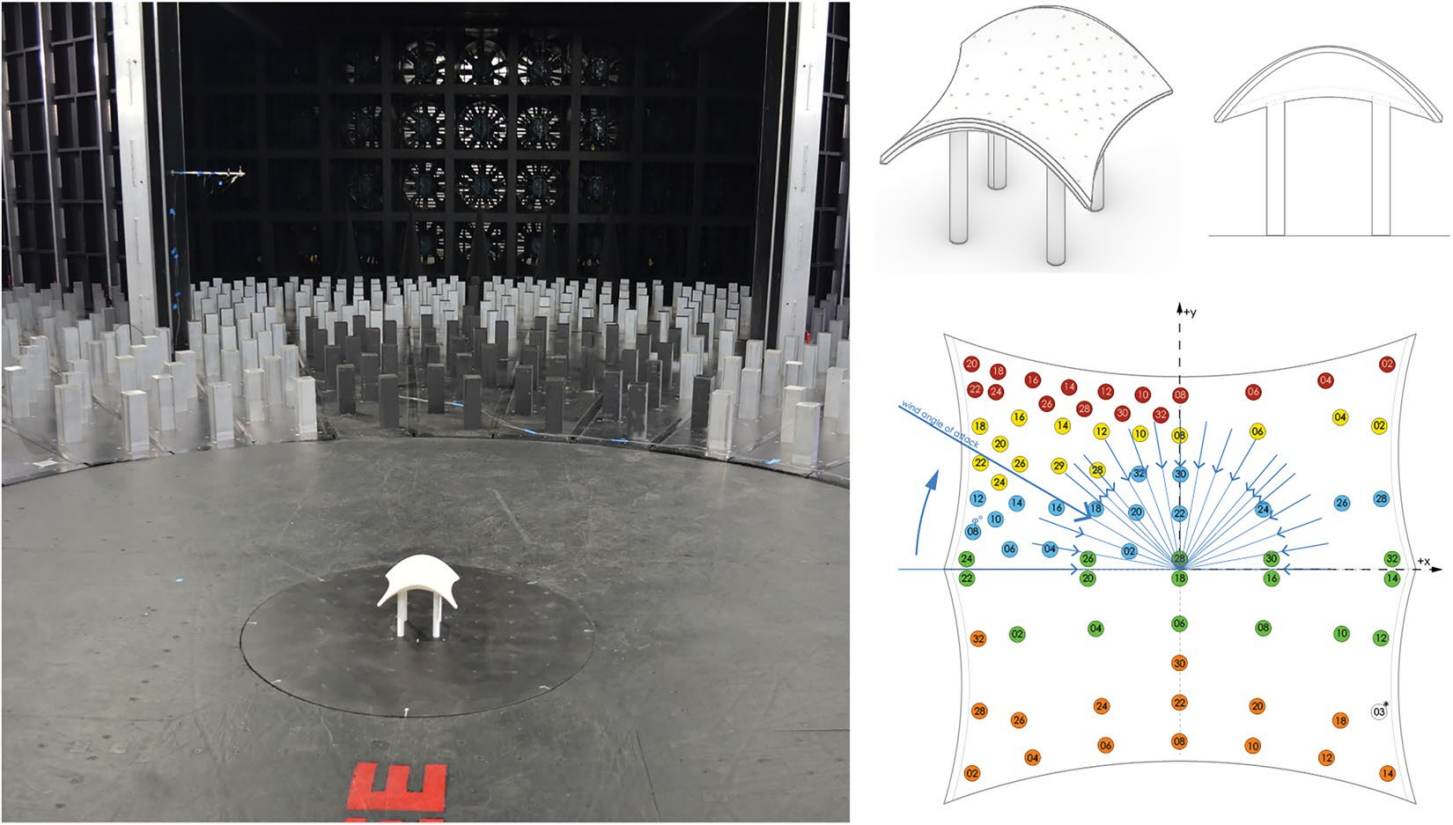
Arch supported (M3) sample tap layout, 3D image and elevation (colors represent scanner numbers - further details available at^8^).

The model was instrumented with approximately 150 pressure taps as shown in Fig. 14. Pressure scanners were connected to the instrumented tap locations on the membrane structure. Pressure tap coordinates are available to correlate instantaneous measurements with locations covering the model in the data set. The taps are evenly distributed across the top and bottom of the entire model with extra taps located on half of the leading-edge quadrant of the model (0° to 90°) to gather additional data along the symmetry plane. The top and bottom taps are directly above/below one another in the z direction, with even tap numbers on top and odd numbers on the bottom following the same order. Upstream velocity measurements were captured using one ‘Straight’ TFI Cobra Probe.

##### ABL loading

This test involved the model described above subjected to low-speed ABL, and high-speed ABL. The model was tested every 10° between 0 and 180° plus a 45° angle of attack.

#### Cone (M4) Pressure Model

**Fig. 15:**
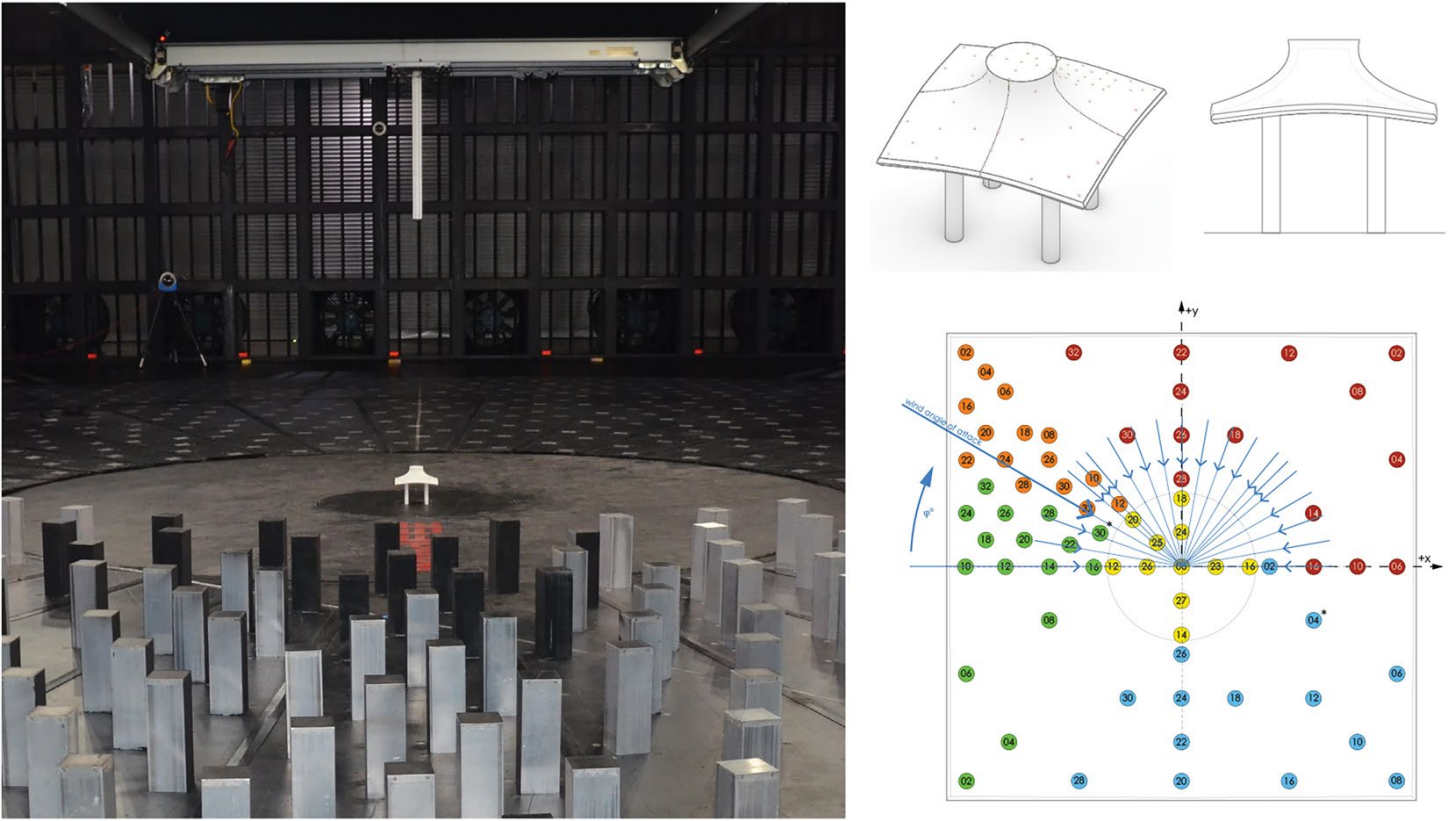
Cone (M4) sample tap layout, 3D image and elevation (colors represent scanner numbers - further details available at^8^).

The purpose of this specimen was to test the Cone (M4) doubly curved geometry under ABL winds in an isolated, 1 × 3 row array, and a 3 × 3 square array configuration. The equilibrium shape of the M4 can be determined via formfinding for an isotropic membrane prestress, with fixed supports at top and bottom circles. The support circle radii are 0.8m and 4.24 m (full-scale). The formfound shapes are then intersected at a 6 m distance in order to generate straight edges for closed array geometries. The full-scale size of the isolated geometry is 6 × 6 × 5 m and at a scale of 1:25 the tested model is 24 × 24 × 20 cm where the canopy itself is 8 cm tall beginning 12 cm from the ground. The canopy is 1 cm thick overall (offset 0.5 cm above and below the original surface geometry) with a 0.6 cm cavity in between for pressure tubing. The model was instrumented with approximately 155 pressure taps as shown in Fig. 15. Pressure scanners were connected to the instrumented tap locations on the membrane structure. Pressure tap coordinates are available to correlate instantaneous measurements with locations covering the model. The taps are generally evenly distributed across the top and bottom (more taps are on the top surface to cover the vertical portion) of the entire model with extra taps located on half of the leading-edge quadrant of the model (0° to 45°) to gather extra data along the symmetry plane. The top and bottom taps are directly above/below one another in the z direction, with even tap numbers on top and odd numbers on the bottom following the same order. Upstream velocity measurements were captured using one ‘Straight’ TFI Cobra Probe.

##### ABL loading - stand-alone

This test involved the model described above subjected to low-speed ABL, and high-speed ABL. The model was tested every 10° between 0 and 180° plus a 45°, 135° angle of attack.

##### ABL loading - row (1 × 3) array

**Fig. 16:**
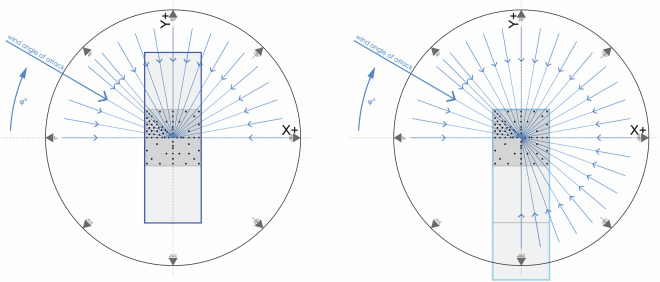
1 × 3 Cone (M4) array configurations with instrumented model at Center (left) and at the corner (right).

This test involved the model described stand-alone, plus two adjacent dummy models arranged in 2 different configurations, subjected to low-speed ABL and high-speed ABL. The configurations include of a 1 × 3 line relative to the main pressure model as shown in Fig. 16. In the configurations, the pressure model is located at the center of the line and at the corner of the line. The model was tested every 10° between 0 and 180° plus a 45° angle of attack.

##### ABL loading - square (3 × 3) array

**Fig. 17:**
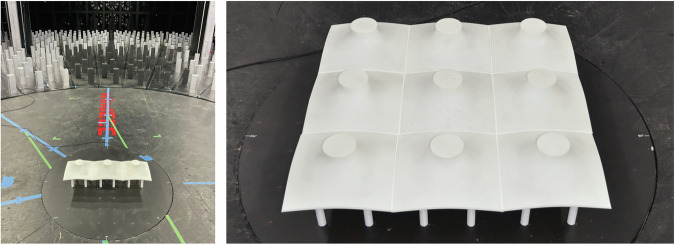
Details of the array testing of cone 1 × 3 array (left) and 3 × 3 array (right).

This test involved the model described in stand-alone plus eight adjacent non-instrumented models arranged in 3 different configurations subjected to ABL wind. The configurations include different locations of the main pressure model within a 3 × 3 square array as shown in Fig. 17. In the configurations, the pressure model is located: at the center of the grid, at the corner of the grid, and at the center edge of the grid. The model was tested every 10° between 0 and 180° plus a 45° angle of attack.

#### Umbrella (M5) pressure model

The purpose of this specimen was to test the Umbrella (M5) doubly curved geometry at a scale of 1:25 under ABL winds. The equilibrium shape of the M5 can be determined via formfinding for a prestress ratio of 4 (e.g. kN/m) as an isotropic membrane prestress to 30 (e.g. kN) in the edge cables, with fixed line supports at the bottom circle (radius of 0.8 m in full scale) and point supports at the top corners. The full-scale size of the geometry is 6 × 6 × 5 m and at a scale of 1:25 the tested model is 24 x 24 x 20 cm where the canopy itself is 8 cm tall beginning 12 cm from the ground. The canopy is 1 cm thick overall (offset 0.5 cm above and below the original surface geometry) with a 0.6 cm cavity in between for pressure tubing.

**Fig. 18:**
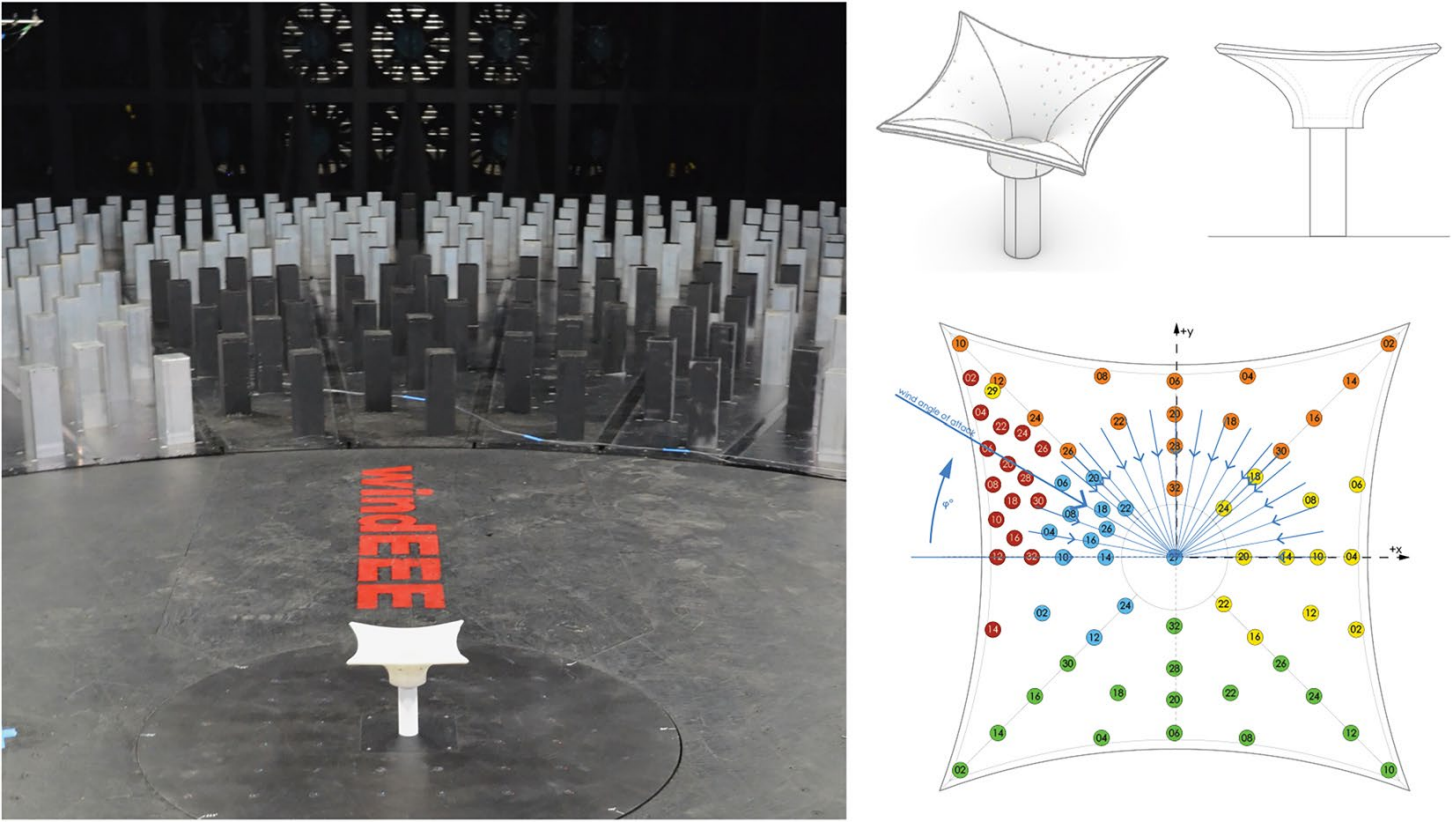
Umbrella (M5) Sample tap layout, 3D image and elevation (colors represent scanner numbers - further details available at^8^).

The model was instrumented with approximately 157 pressure taps as shown in Fig. 18. Pressure scanners were connected to the instrumented tap locations on the membrane structure. Pressure tap coordinates are available to correlate instantaneous measurements with locations covering the model. The taps are generally evenly distributed across the top and bottom (more taps are on the top surface to cover the vertical portion) of the entire model with extra taps located on half of the leading-edge quadrant of the model (0° to 45°) to gather extra data along the symmetry plane. The top and bottom taps are directly above/below one another in the z direction, with even tap numbers on top and odd numbers on the bottom following the same order. Upstream velocity measurements were captured using one ‘Straight’ TFI Cobra Probe.

##### ABL loading

This test involved the model described above subjected to low-speed ABL, and high-speed ABL. The model was tested every 10° between 0 and 180° plus 45°, 135° angle of attack.

## Data Records

**Fig. 19:**
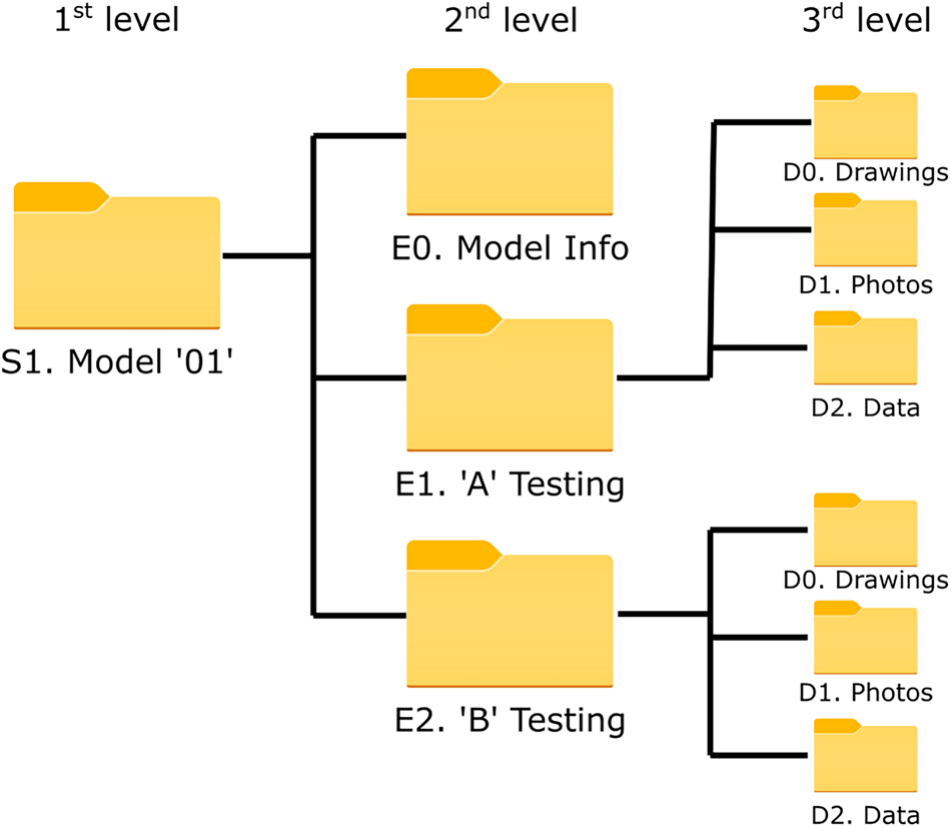
Folder structure of the dataset in^8^.).

The presented dataset of measurement data is made available in zenodo as Bletzinger *et al*.^8^. To support ease of use and traceability, the data and accompanying documentation are organized into a structured folder hierarchy. This is categorized by test model/specimen, experiment type, and content type, as summarized in Fig. 19. The top-level directories include the five models tested, as well as documentation and ABL profile development information. In each of the model folders, general model information (E0) and separate folders for each wind test scenario are present. Each test folder contains standardized subdirectories–D0 for drawings, D1 for photos, and D2 for data–to systematically store associated materials.

The main data folders available in the repository are:

- **S0. Documentation**: Includes the specification of instruments, details of the WindEEE Flow description, and an Excel file containing file names of all the tests with details (WENSS-Test Program-Zenodo.xlsx). This Excel file provides a master reference for navigating and interpreting the full dataset.
- **S1. Profile Development**: Includes ABL profile development and validation data.
- **S2. M1 (Hyparbolic Parabloid Pressure Model**: Contains pressure data for the hypar model tested under ABL, downburst, and tornado conditions.
- **S3. M2 (Ridge Valley) Pressure Model**: Contains pressure data for the ridge valley model tested under ABL conditions.
- **S4. M3 (Arch Supported) Pressure Model**: Contains pressure data for the arch supported model.
- **S5. M4 (Cone) Pressure Model**: Includes data for the cone model tested in isolated, 1 × 3, and 3 × 3 configurations.
- **S6. M5 (Umbrella) Pressure Model**: Contains pressure data for the umbrella model.

Each data file follows a structured naming convention that encodes key metadata about the experiment. Table 1 describes the naming convention followed. Each file name component encodes key metadata about the test scenario, including the project identifier, model type, wind profile, orientation angle, configuration, and a run number. This format ensures consistency and traceability across experiments and models, enabling users to easily identify and organize data associated with specific test cases. Details of wind profiles and configurations are cross-referenced with the ‘S0. Documentation\WENSS-Test Program-Zenodo.xlsx’ in the dataset.

**Table 1:** File naming convention followed in the dataset^8^.

Parameter	Example	Description
**Base File Name**	WNS-MX-CC-0000-A315-C01-R00	The file name can be broken down to describe the test case it represents such that: WNS = Project Name MX = Model ID CC-0000 = Unique WindEEE Profile A000 = Model Global Angle C01 = Model configuration R00 = Run # ID
**Profile**	CC-0000	This example would be recognized as a zero measurement. However, the example ‘CC-3045’ represents a unique WindEEE profile, details are available in the profile development folder.
**Angle**	A315	Represents a global model angle of 315 degrees to the 60-fan wall (refer to WENSS-Test Program-Zenodo.xlsx for specifics)
**Config**	C01	Represents the matching configuration in the WENSS-Test Program-Zenodo.xlsx provided. A specific configuration is a combination of model placement in the wind tunnel and wind scenario considered (ABL, downburst, or tornado)
**Run**	R00	Represents a zero measurement. However, ‘R01’ would represent the first test run of a specific configuration.

For efficient storage, ease of access, and increased compatibility, all data files in this repository are using the *.mat MATLAB v6 (version 6) format. The *.mat format is accessible not only through MATLAB, but also supported by most programming languages preferred within the scientific community, like native support in MATLAB, scipi.io in Python, R.matlab package in R, built-in load and save commands in GNU Octave, matio library for C/C++, JMatIO library for Java, and MAT.jl package in Julia.

## Technical Validation

The technical validation of the data set is done by validation of the measurement instruments, validation of the generated ABL wind profile, and validation of the WindEEE facility. Cobra probes were used to measure the pressure data. Chen *et al*.^12^ validated the use of Cobra probes for turbulence measurement by comparing their output against established data for fully developed pipe flow. The study showed good agreement, confirming the probe’s capability to capture higher-order turbulence statistics accurately. Mallipudi *et al*.^13^ evaluated the performance of Cobra probe for measuring unsteady 3D velocity fields in turbulent wake flows. The study included an in-situ calibration, which showed good agreement with manufacturer specifications.

Figure 6 presents the vertical profiles of mean wind speed and longitudinal turbulence intensity (TI) used in the ABL flow. The spectral distribution of longitudinal wind fluctuations at the mean roof height of the tested pressure models is shown on the right. The mean wind profile, turbulence intensity, and spectral characteristics are validated against the requirements of the ASCE wind loading provisions.

The effectiveness and reliability of the experimental procedures used to simulate non-stationary winds at the WindEEE Dome have been extensively validated over the past years. Direct comparisons between full-scale downburst events and their WindEEE Dome counterparts are presented in several previous studies^14–16^. Tornadic flows have also been studied and validated at the windEEE facility^11,17–19^. The obtained pressure measurements were post-processed to compute the net pressure coefficients using Eq. (1). The resulting mean pressure coefficient distributions are presented in Fig. 21. These results are comparable to the coefficient of pressure plots shown in Fig. 9 of^20^, demonstrating similar spatial patterns and magnitudes across the tested geometries.

## Usage Notes

To analyze the results from the project, future users of the dataset will inevitably need to convert pressure tap data into something more visual, like a pressure distribution plot. This involves two important steps. First, the resultant pressure acting on the membrane consists of both top and bottom pressures combined. It is then necessary to couple each data point with the corresponding one from the opposite side of the membrane. Second, the data stored in points will need to be somehow mapped or interpolated into the original surface to generate pressure distribution plots.

This section presents a brief overview of the initial analysis of the experimental data set presented on the five representative doubly curved membrane structures. These figures demonstrate potential applications of the data set presented. While over 425 tests were conducted across various wind conditions and angles of attack, the results shown here are intended to highlight key trends and distinctive aerodynamic behaviors observed across the dataset. Tests were conducted under Atmospheric Boundary Layer (ABL) flow, with additional tornado and downburst scenarios for the hypar geometry. The results are summarized in terms of mean pressure distributions, pressure time histories, and flow field characteristics.

The pressure data obtained from wind tunnel experiments are shown directly in Pascal. Nevertheless, the non-dimensional pressure coefficient, *C*_*p*_, is used to compare different models of different scales, defined in Eq. (1).

1 $${C}_{p}=\frac{p-{p}_{\infty }}{\frac{1}{2}\rho {V}^{2}}$$

where *p* is the local surface pressure, *p*_*∞*_ is the free-stream pressure, *ρ* is the air density, and *V* is the reference wind speed. This approach can reconstruct pressure distributions on full-scale structures from wind tunnel measurements. Although achieving strict Reynolds number similarity is often challenging, the experimental setup was designed to minimise its influence on the pressure field. Where necessary, deviations due to Reynolds number effects can be estimated using numerical simulations or empirical correction factors. Overall, the methodology presented is suitable for application across a wide range of structural scales, making it a practical tool for evaluating wind-induced pressures beyond the confines of the tunnel model.

**Fig. 20:**
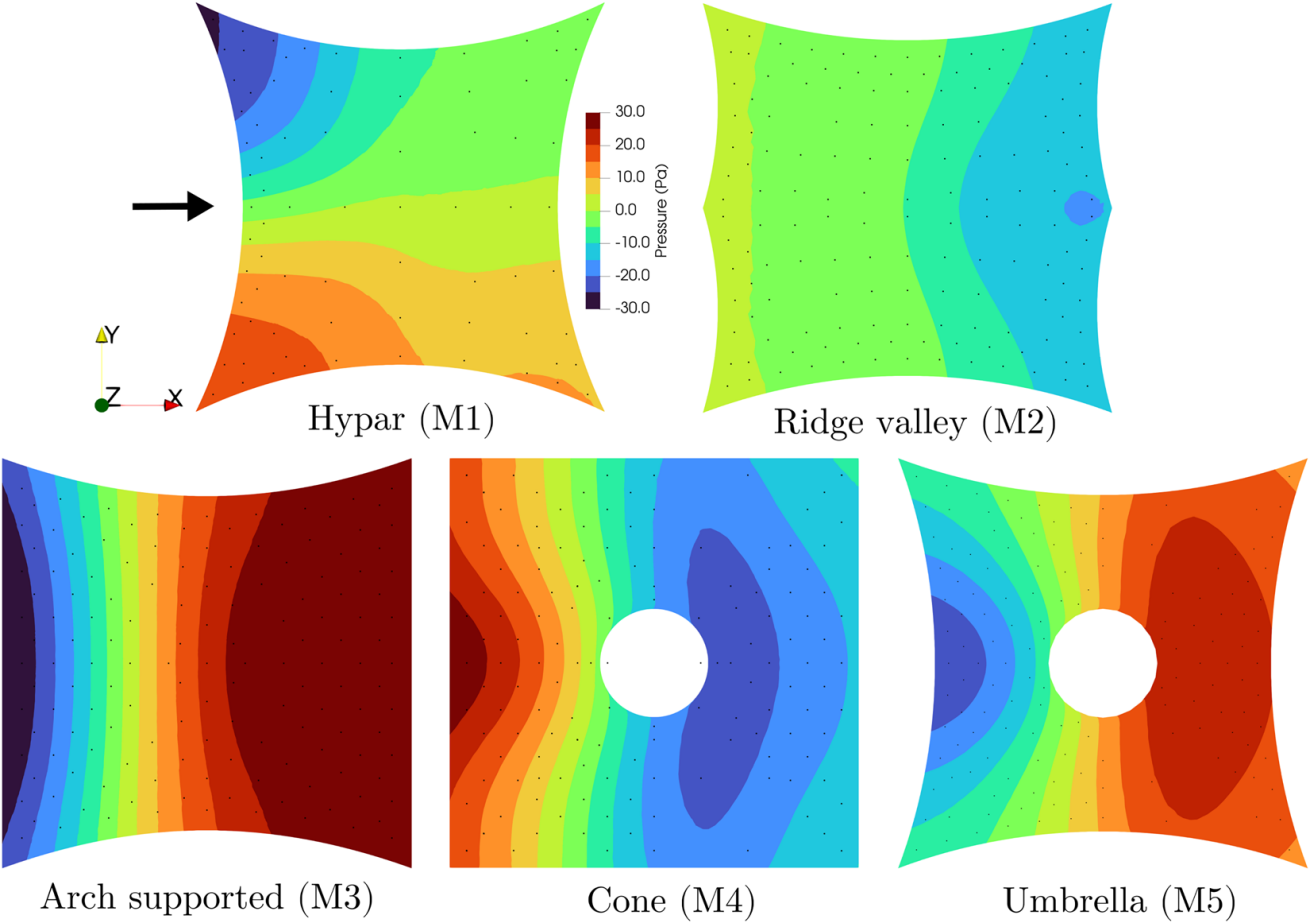
Mean pressure plots for 0^°^ incoming wind for ABL flow of each model.

**Fig. 21:**
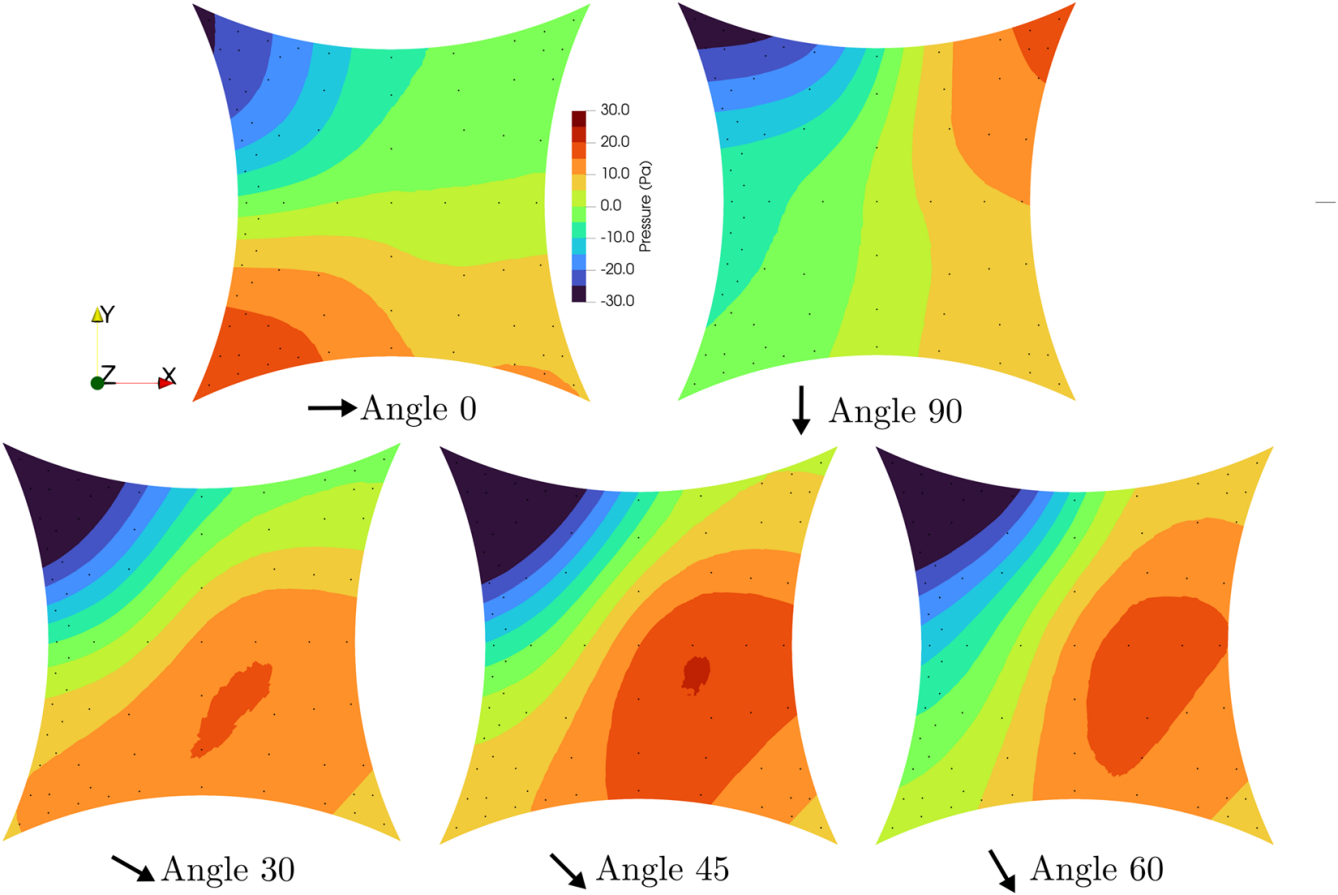
Mean pressure plots for multiple angles of incoming wind for ABL flow of the Hypar model.

Five geometries, hypar (M1), ridge valley (M2), arch supported (M3), cone (M4), and umbrella (M5), are presented, and the mean net pressure distribution under ABL flow for zero degrees angle of attack is shown in Fig. 20. The net pressure is computed as the difference between the top and the bottom pressures. A positive net pressure indicates a downward resultant. Black dots represent the pressure tap locations. The figure highlights how the pressure changes with respect to the shape. As a representative geometry, hypar (M1) is shown under multiple wind angles of attack in Fig. 21. The figure shows how the mean net pressure changes for various angles of attack.

**Fig. 22:**
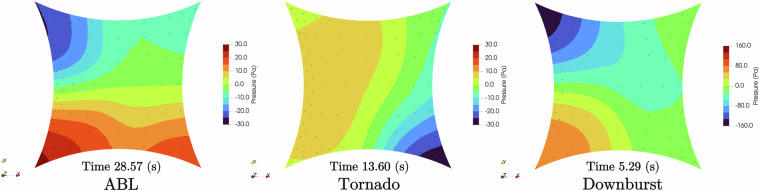
Pressure plots corresponding to the maximum time instant of the Hypar model in Fig. 7 for all the wind scenarios.

For the hypar (M1) geometry, three wind scenarios were tested: ABL, tornado, and downburst. The time histories of the velocity components in the *u*, *v*, and *w* directions–representing wind velocity in the *x*, *y*, and *z* axes, respectively–are presented in Fig. 7. These profiles illustrate the characteristic flow behavior of each wind scenario. The peak velocity in the x-direction is highlighted with a green circle, marking the time step of maximum dynamic impact in the streamwise direction. The corresponding instantaneous pressure distributions on the Hypar surface at these peak time steps are shown in Fig. 22, providing insight into the spatial pressure response under each of these flow conditions.

**Fig. 23:**
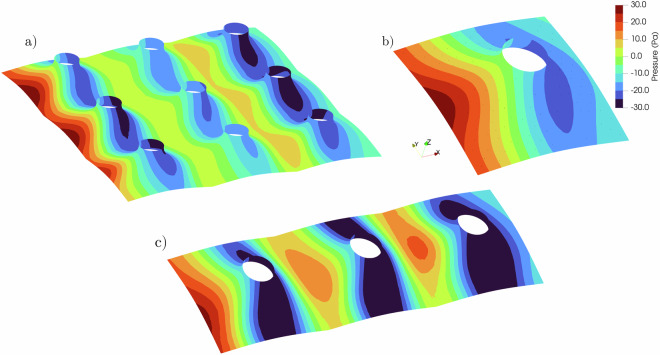
Mean pressure plots of the single, 1 × 3 and 3 × 3 arrays for the Cone (M4) geometry under ABL wind 0° anglewith (**a**) 3 × 3, (**b**) isolated, and (**c**) 1 × 3 configurations.

**Fig. 24:**
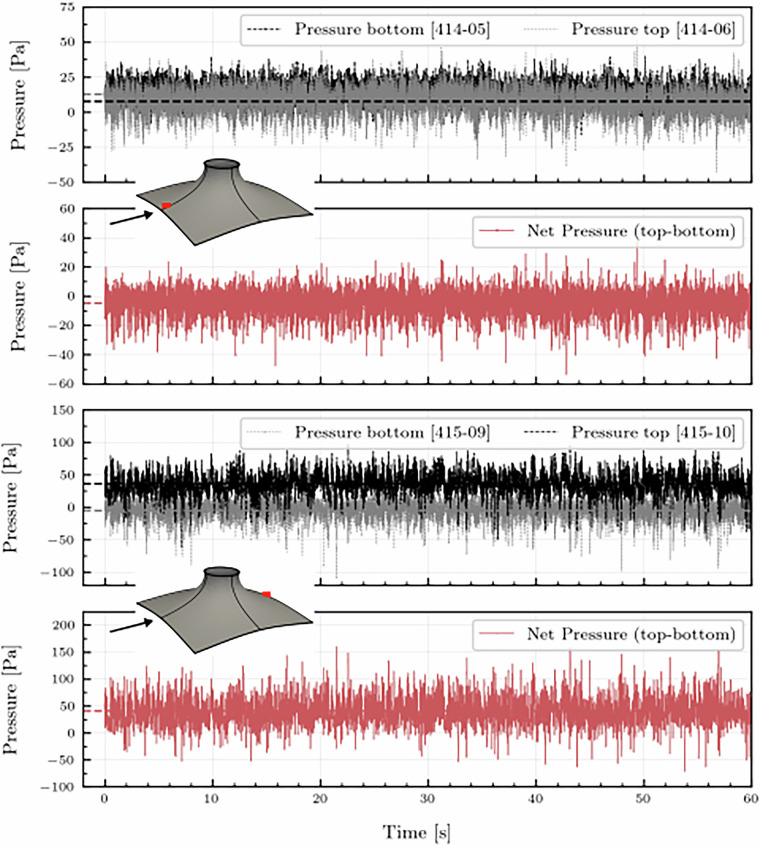
Pressure vs Time for top and bottom pressure taps and the net pressure for two pressure taps for the Cone (M4).

The cone (M4) geometry was tested in single, 1 × 3, and 3 × 3 array configurations to identify potential effects due to neighboring structures. Figure 23 presents the mean net pressure distributions for the three configurations. Figure 24 shows the time series of top and bottom surface pressures, along with the resulting net pressure at two selected tap locations on the model. These comparisons illustrate the fluctuations in pressure and how the net pressure is computed.

It is recommended that the provided Python file in^21^ be used to combine the top and bottom pressure taps. The Python file is an example of the hypar case. It can be used as a template for other geometries. It is recommended that Paraview be used to carry out the second part of this process since it provides useful tools to map point data results into surfaces. There is no definitive answer to which exact method provides the best results - every interpolation method is still an approximation based on the original data. Point Interpolation filter was used to map point data into an imported surface. An ellipsoidal Gaussian kernel was selected to interpolate pressure values. The plots shown in Figs. 20–23 are with EllipsoidalGaussianKernel having a Radius of 100, Sharpness of 3, and an Eccentricity of 2.

## Data Availability

The code used for processing the data is in Python and is available at^21^.
